# Roles of Endophytic Fungi Isolated from *Mangifera indica* L. in Promoting Plant Growth

**DOI:** 10.4014/jmb.2401.01034

**Published:** 2024-07-19

**Authors:** Kanyapat Sonsiam, Orlavanh Sonesouphap, Anyaporn Sangkaew, Pariyanuj Chulaka, Prakitsin Sihanonth, Chulee Yompakdee

**Affiliations:** 1 Department of Microbiology, Faculty of Science, Chulalongkorn University, Bangkok 10330, Thailand; 2 Department of Horticulture, Faculty of Agriculture, Kasetsart University, Chatuchak, Bangkok10900, Thailand

**Keywords:** Endophytic fungi, plant growth promoter, nutrients solubilization, *Aureobasidium pullulans*, *Aspergillus tamarii*

## Abstract

Endophytic fungi have been shown to synthesize bioactive secondary metabolites, some of which promote plant growth through various mechanisms. In our previous study, endophytic fungi were isolated from mango trees (*Mangifera indica* L.). The present study examined fifty endophytic fungal isolates for mineral solubilization activity, ammonia production, and siderophore production. It was shown that these isolates could produce phytohormones indole-3-acetic acid and gibberellic acid, as well as inhibit plant pathogens, specifically *Colletotrichum gloeosporioides* and *Lasiodiplodia theobromae*. The results showed that all the isolated fungal endophytes exhibited various activities. Based on the findings, two fungal endophytes—*Aureobasidium pullulans* CY.OS 13 and *Aspergillus tamarii* CY.OS 144—were selected for dual inoculation in chili plants under pot-scale conditions to investigate their potential to improve growth-related traits such as seed germination, shoot and root length, biomass, and chlorophyll content. Seed treated with *A. pullulans* CY.OS 13 and/or *A. tamarii* CY.OS 144 showed a significant (*p* < 0.05) increase in seed germination and growth parameters of chili plants grown under pot-scale conditions. Particularly, chili plants whose seeds were injected with a combination of the two selected endophytic fungi showed the highest plant development traits. Therefore, the selected endophytic fungi have the potential to be used as biofertilizers, especially when combined. They could eventually replace chemical fertilizers because they are environmentally friendly, beneficial to humans, and can even promote sustainable agriculture.

## Introduction

The increasing global population's demand for food is predicted to drive agricultural development. The demand is estimated to rise to 8.5 billion people (a 10% increase) in 2030 and 9.7 billion people (a 26% increase) in 2100 [1, 2]. Annually, numerous agricultural crops are consumed and exported domestically and internationally, especially economic crops such as chili [3]. Farmers use chemical fertilizers, growth hormones, and pesticides to enhance the productivity of agricultural crops in response to consumer demand, as these methods are efficient and yield fast results. However, the cost of fertilizers and crops has also increased. Chemical fertilizers also have an impact on the soil, plants, and the health of consumers in the cultivation area. As a result of the harmful impacts of conventional fertilizers, increasing consumer demand, and health concerns, most farmers are interested in "organic farming," which involves the use of biofertilizers to promote plant development.

Endophytic fungi colonize the internal healthy plant tissues, such as leaves, stems, twigs, bark, roots, fruits, flowers, and seeds, without causing harm or pathogenic infection to host plants [4]. These endophytes are found in almost all plant species and are usually beneficial to the host. Through plant-endophyte interaction, they produce bioactive secondary metabolites, which are valuable natural resources with a wide range of applications [5-7]. In terms of agricultural applications, some endophytic fungi isolated from various plant species have been reported to promote plant growth through direct and indirect mechanisms. Thus, the release and mobilization of insoluble nutrients is important for improving soil nutrient availability to the crop. Recently, it has been reported that endophytic fungi isolated from various plant species enhance plant growth by producing and/or solubilizing plant nutrients. Some endophytes produce ammonia (NH_3_) as a nitrogen source and exhibit nutrient-solubilizing activity to support plant growth [8-13]. Under iron-limiting conditions, endophytic fungi produce iron-chelating siderophores that enhance plant growth by facilitating iron (Fe) uptake and preventing infection by fungal pathogens [14-17]. There have been reports on the use of microbial combinations to promote plant growth. For example, the combination of endophytic bacteria *Bacillus subtilis* and rhizobacteria *Pseudomonas fluorescens* showed synergistic interactions as biocontrol agent [18]. Therefore, the combination of endophytes can be used to promote plant growth and development, especially in important crop species.

In a previous study, we isolated endophytic fungi from mango trees (*Mangifera indica* L.) that exhibited potential for phytohormone production and antifungal activity against phytopathogens. Among these, the endophytic yeast *Aureobasidium pullulans* CY.OS 13 exhibited the highest activity in phytohormone production, which is crucial for promoting plant growth [19]. However, the plant-promoting potential of endophytic fungi and their effect on promoting plant growth under pot-scale conditions have not been determined. Therefore, the aim of the present study was to evaluate the impact of endophytic fungal isolates on plant-promoting activities, such as nutrient solubilization and production. The impact of two selected types of endophytic fungi on the growth of chili plants under pot-scale conditions was also evaluated.

## Materials and Methods

### Source of Endophytic Fungi

Fifty endophytic fungi used in this study were obtained from Ms. Orlavanh Sonesouphap [19] (Table 1). In April and May 2014, endophytic fungi were isolated from various parts of three cultivars of *M. indica* L. (mango) collected from Phontan village in Saysattha district, the capital of Vientiane, Laos (17°57'N, 102°38'E). They comprised 37 endophytic molds and 13 endophytic yeasts, and were selected based on their ability to produce phytohormones (both indole-3-acetic acid (IAA) and gibberellic acid (GA3)) and/or exhibit antifungal activity against two fungal phytopathogens viz., *Colletotrichum gloeosporioides* and *Lasiodiplodia theobromae*.

### *In vitro* Screening of Endophytic Fungi for Plant Growth-Promoting Properties

**Phosphate solubilization assay.** The phosphate solubilizing ability of all endophytic fungi was assessed using National Botanical Research Institute’s phosphate growth medium (NBRIP) (per liter: 10 g glucose, 0.1 g (NH_4_)_2_SO_4_, 5 g MgCl_2_·6H_2_O, 0.25 g MgSO_4_·7H_2_O, 0.2 g KCl, 15 g agar and supplemented with 5 g Ca_3_(PO_4_)_2_) [20]. The assay plates were incubated at 30°C for 7 days. A clear halo zone surrounding the fungal colony was measured to estimate the solubilization ability of the endophyte using Eq. (1):

$$\text{solubilization efficiency (SE)=}\frac{\text{clear zone diameter}}{\text{colony diameter}}$$ (1)

**Potassium and zinc solubilization assays.** Screening of endophytic fungi for potassium and zinc solubilizing activity was performed by growing the fungi on fresh Aleksandrov (per liter: 5 g glucose, 0.5 g MgSO_4_·7H_2_O, 0.1 g CaCO_3_, 0.006 g FeCl_3_, 2 g Ca_3_PO_4_, 15 g agar and supplemented with 3 g KAl_2_(AlSi_3_O_10_) (OH)_2_) [21] and zinc oxide agar (per liter: 10 g glucose, 0.5 g yeast extract, 0.1 g MgSO_4_·7H_2_O, 0.5 g (NH_4_)_2_SO_4_, 0.2 g KCl, trace of MnSO_4_ and FeSO_4_, 15 g agar and 12 g ZnO) [22], respectively. The solubilization efficiency was calculated as described above.

**Siderophore production assay.** Siderophore detection was carried out using the universal assay [23]. Endophytes were grown in the center of a Chrome Azurol S (CAS) agar plate. The non-inoculated plate was used as a control. The plates were placed in the dark at 30°C for 7 days and then examined for the presence of an orange halo zone surrounding the fungus on blue-colored agar media. The halo was measured using the same method employed to evaluate phosphate solubilization. The results were expressed as production efficiency (PE).

**Ammonia production assay.** Ammonia production ability was examined using the method described in an earlier study [24] with slight modifications. Briefly, all endophytic fungi were grown for 5 days in peptone water (per liter: 10 g peptone and 5 g NaCl) at 30°C with shaking at 200 rpm. Peptone water without a fungal inoculation served as the control. The culture supernatant was obtained by centrifugation at 4,000 rpm for 10 min. The cell pellet was washed and dried to obtain the total cell dried weight. To determine the amount of ammonia, 0.02 ml of 1.5 M potassium sodium tartrate (KNaC_4_H_4_O_6_) solution and 0.02 ml of Nessler’s reagent were added to 1 ml of the supernatant. After 20 min, the absorbance at a wavelength of 425 nm was measured using a BioMate BioMateTM 3S spectrophotometer (Thermo Scientific, USA) to determine the ammonia concentration. The corresponding absolute calibration curve was prepared using aqueous ammonium chloride (NH_4_Cl) solutions with a set of known values as standards (mg/l) [25, 26]. The results were expressed as ammonia production activity (mg/g DCW).

### Plant Growth-Promoting Effect of Endophytic Fungi on Chili

To investigate the effect of single and combined inoculations of endophytic fungi on plant growth, endophytic yeast *A. pullulans* CY.OS 13 and endophytic mold *Aspergillus tamarii* CY.OS 144 (two strains of non-plant pathogenic endophytic fungi) were employed.

***In vitro* antagonistic effect of two selected endophytic fungi by dual culture method.** To investigate whether the selected endophytic fungi could be used in combination for inoculation, the antagonist activity was determined using the dual culture approach [27, 28]. For the combination of two selected endophytic fungi, a mycelium plug (5 mm in diameter) of *A. tamarii* CY.OS 144 was placed on one side of a YPD plate, while *A. pullulans* CY.OS 13 was streaked on the side of the plate opposite to the mycelium plug. The control plate was inoculated with mycelial plugs only and kept at 30°C for 7 days. The inhibitory effect was determined by measuring the zone of inhibition.

**Impact of endophytic fungi on chili seed germination using top-of-paper (TP) method.** The potential of fungal endophytes as plant growth promoters was assessed by studying seed germination using the TP method [29]. The experimental design was conducted using a completely randomized design (CRD) with four (4) replicates for each treatment (N = 400 per treatment), and the selected fungal endophytes were prepared for inoculation. Chili seeds were disinfected using 70% ethanol (v/v) for 1 min, 5% sodium hypochlorite (v/v) for 5 min, and again in 70% ethanol (v/v) for 1 min, then rinsed 3-5 times in sterile distilled water [30]. The surface-sterilized seeds were immersed in endophytic fungi suspension for 3 h, air-dried, and placed in petri dishes on three layers of moistened Whatman filter paper no.1. The plate was sealed with plastic wrap and incubated at 28°C for 6-14 days. The percentage of seed germination was determined using Eq. (2):

$$\text{Percentage of seed germination (\%) =}\left(\frac{\text{Number of seed germinated}}{\text{Total number of seeds}}\right)\text{×100}$$ (2)

**Effect of endophytic fungi on promoting chili growth under pot-scale condition.** The ability of the selected fungal endophyte was tested in chili plants using the method developed in previous studies with slight modifications [29, 31]. Briefly, pots were sterilized with 20% sodium hypochlorite (v/v) and filled with sterile compost soil (Sudaratkrankasret Inc., Thailand). Thereafter, chili seeds were immersed in the designated concentrations of endophytic suspension. The cells or spores suspension was counted using a hemocytometer and adjusted to the final concentration. There were six treatments in this experiment: (T1) seeds treated with sterile-distilled water as a control, (T2) seeds treated with 1 × 10^5^ cells/ml of *A. pullulans* CY.OS 13, (T3) seeds treated with 1 × 10^8^ cells/ml of *A. pullulans* CY.OS 13, (T4) seeds treated with 1 × 10^5^ spores/ml of *A. tamarii* CY.OS 144,(T5) seeds treated with 1 × 10^8^ spores/ml of *A. tamarii* CY.OS 144, and (T6) seeds treated with the combination of 1×10^8^ cells/ml of *A. pullulans* CY.OS 13 and 1 × 10^8^ spores/ml of *A. tamarii* CY.OS 144. The chili seeds were then sown in plastic pots filled with 2 kg of sterile soil. On the 14th day after sowing, the chili seedlings were thinned to one plant per hole. The pots were arranged in a completely randomized factorial design. The seedlings were grown in pots placed in an area covered with insect netting, exposed to a day/night cycle of 13-14 h natural light. Four weeks after seedling emergence, the chili plants (10 plants per treatment) were harvested, cleaned and examined for their morphological characteristics, such as shoot and root length, fresh and dry weight, as well as the levels of chlorophyll A and B in each plant.

Chlorophyll A and B contents were determined using established techniques as described in a previous study[32]. Briefly, chili leaves were cut into small pieces, weighed, and extracted with 80% acetone. The extract was measured at wavelengths of 663 nm (chlorophyll A), and 645 nm (chlorophyll B) using a spectrophotometer. The chlorophyll contents were expressed as mg/g FW and calculated using Eqs. (3) and (4):

$$\text{Chlorophyll A=}\frac{\text{[12.25(OD663)-2.79(OD645)]}}{\text{1000×FW}}\text{×V}$$ (3)

$$\text{Chlorophyll B=}\frac{\text{[21.5(OD645)-5.10(OD663)]}}{\text{1000×FW}}\text{×V}$$ (4)

where FW is the fresh weight (g) and V is the extraction volume (ml).

All experiments were performed in triplicate (N= 30/treatment).

### Determining the Colonization of the Selected Endophytic Fungi in Chili Plants

The colonization of inoculated endophytic fungi into chili plant was determined by re-isolating the endophytic fungi from plant tissues, identifying the isolates, and comparing them with the inoculated strains using Polymerase Chain Reaction-Restriction Fragment Length Polymorphism (PCR-RFLP) assay. The root sections of the chili plants inoculated with endophytic fungi were collected and aseptically cut into small pieces (ca. 3 cm). The seeds were then surface sterilized using the same method described above prior to cultivation. To cultivate endophytic fungi, segments of plant samples were placed aseptically on potato dextrose agar (PDA) and yeast malt agar (YM; per liter: 3 g yeast extract, 3 g malt extract, 5 g peptone, 10 g glucose, and 15 g agar) supplemented with antibiotics (0.1 mg/ml ampicillin and 0.1 mg/ml kanamycin). This was done to prevent bacterial contamination. The endophytic fungi that emerged from the plant segments were then subcultured onto new media until a pure culture was achieved.

The identification of endophytic fungi was based on morphological and molecular analysis. After grouping the morphological traits, genomic DNA of endophytic yeast was extracted using the CTAB method [33]. Subsequently, the D1/D2 region of the large subunit of the rRNA gene was amplified with a pair of universal primers, NL1 (5’ GCATATCAATAAGCGGAGGAAAAG 3’) and NL4 (5’ GGTCCGTGTTTCAAGACGG 3’). Using a modified glass bead method [34], genomic DNA was isolated from the endophytic mold. Thus, the internal transcribed spacer (ITS) region of ribosomal DNA (rDNA) was amplified using a pair of universal primers, ITS1 (5’ TCCGTAGGTGAACCTGCGG 3’) and ITS4 (5’ TCCTCCGCTTATTGATATGC 3’). The PCR products were examined on a 1% agarose gel and purified for restriction fragment length polymorphism (RFLP) analysis. Three restriction enzymes, namely *Hae*III, *Hin*fI and *Hha*I, were utilized following the manufacturer's instructions (NEB, England). After digestion, fragments were separated using gel electrophoresis in 2% agarose at 80 V for 2 h. The restriction fragment profiles of each strain were compared.

### Statistical Analysis

Statistical analyses were performed using Microsoft Office Excel 2010 and GraphPad Prism 5.01 programs. One-way analysis of variance with Tukey's test was used to assess the significance of differences between pairs of group means. All experiments were conducted using a completely randomized design, with 3-4 replications. At least three independent experiments were performed. Data presented are from a representative experiment.

## Results and Discussion

### Isolated Endophytic Fungi Exhibiting Plant Growth Promoting Activities

**Fig. 1:**
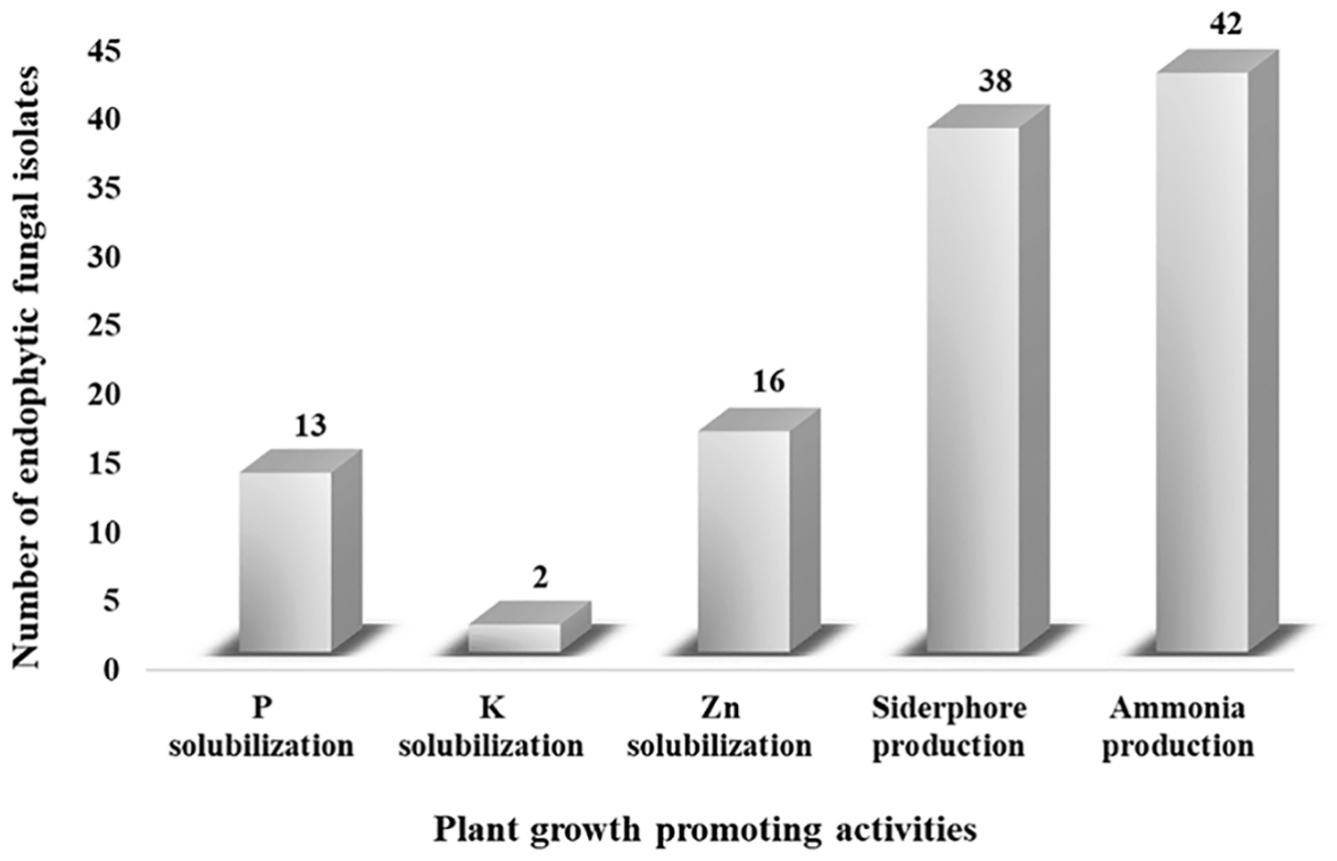
Number of endophytic fungi isolate exhibiting plant growth promoting activities. Their ability in solubilization of phosphate (P), potassium (K), and zinc (Zn) as well as production of phytohormone (that is, siderophore) and ammonia are shown.

Microorganisms from various sources can recycle nutrients and enhance plant growth. Therefore, microorganisms with various plant growth-promoting properties have emerged as an important and effective tool for improving agricultural output [1, 35-37]. Here, all endophytic fungi isolates (Table S1) were evaluated for plant growth-promoting properties, including the activity of phosphate, potassium and zinc solubilizations, siderophore production, and ammonia production (as shown in Fig. 1).

**Phosphate, potassium, and zinc solubilizations.** One of the main mechanisms of plant growth involves the solubilization of insoluble minerals by microorganisms. Phosphate is an essential nutrient for plant growth and development, playing a crucial role in processes such as photosynthesis, sugar production, and energy generation. It is also involved in all metabolic processes [38]. In addition, phosphate-solubilizing microorganisms are known to minimize the release of inorganic phosphate and improve soil fertility [39]. To determine the phosphate-solubilizing capacity of fungal colonies, the NBRIP agar medium assay plate was used, which is more efficient than the Pikovskaya medium (PVK) [20]. Out of the 50 endophytic fungal isolates tested (Table S1), 13 were found to be effective in this activity as they produced clear halos surrounding the colony on NBRIP medium (Fig. 1), indicating a phosphate-solubilizing ability. The highest phosphate solubilization efficiency was observed for the endophytic yeast *Candida* sp. CY.OS 07 (1.61 ± 0.19).

To assess the endophytic isolates' ability to solubilize potassium and zinc, Alexanderova agar and zinc oxide agar were utilized. Based on our findings, *Candida* sp. CY.OS 07 and *Penicillium citrinum* CY.OS 145 showed the highest solubilization efficiencies of 1.38 ± 0.04 and 2.17 ± 0.12 for potassium and zinc, respectively.

**Siderophore and ammonia production.** Siderophores are secondary metabolites that chelate iron and are produced by a variety of microorganisms. They are useful for scavenging iron-limited environments. By supplying plants with iron, siderophores produced by endophytes promote plant growth [40]. Through iron depletion, siderophores can have a negative effect on pathogenic fungi [41]. In this study, all endophytic fungal isolates (Table S1) were cultured on CAS agar in order to examine the siderophore-producing endophytes. Out of the 50 isolates, 38 were found to produce siderophores (Fig. 1). The results showed that the endophytic mold, *P. citrinum* CY.OS 185, had the highest siderophore production efficiency values (4.60 ± 0.30).

**Fig. 2:**
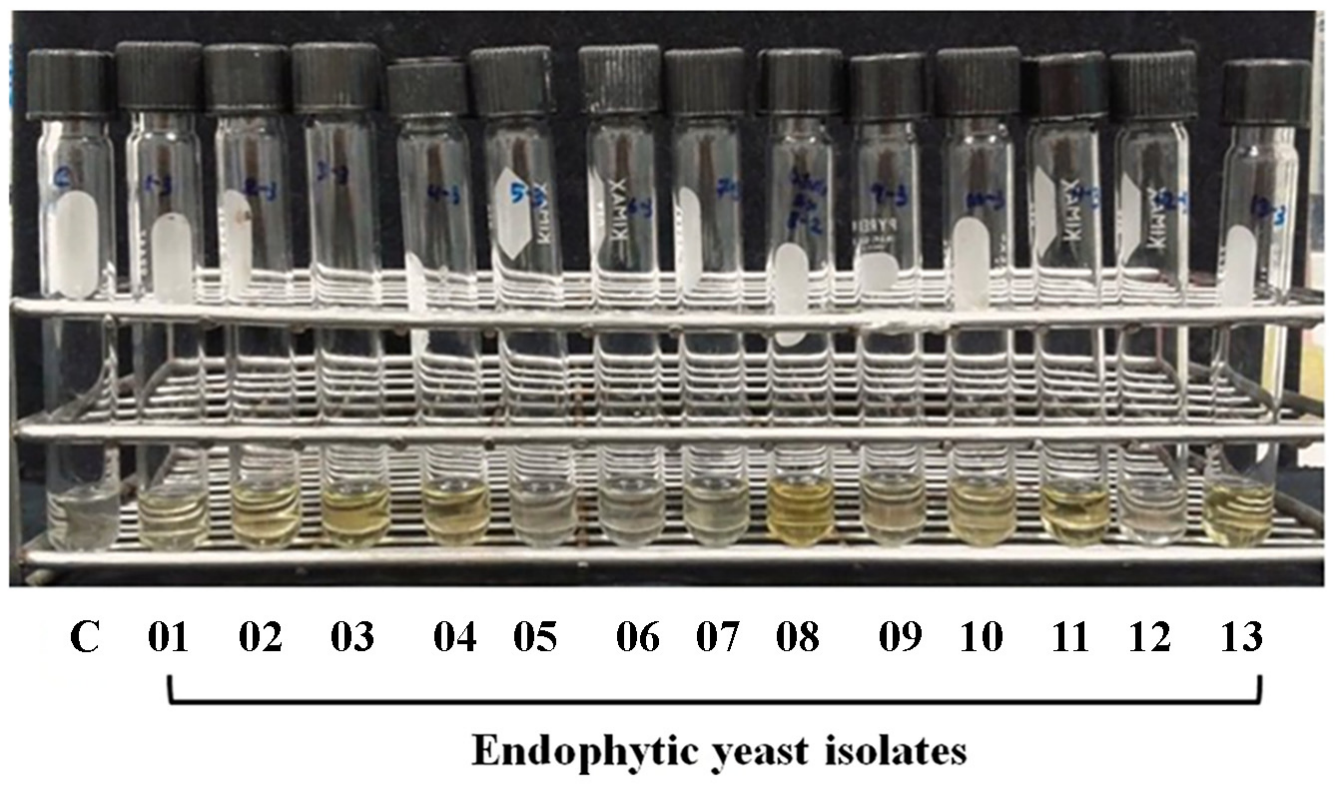
Ammonia-producing activity of the endophytic fungi. Ammonia production activity of the fungal endophyte isolates CY.OS 01, 02, 03, 04, 07, 08, 09, 10, 11 and 13 is detected using Nessler’s reagent method. The control (C) is peptone water containing Nessler’s reagent.

**Ammonia production assay.** The ability of plant growth-promoting endophytes to produce ammonia is one of the mechanisms that support the growth and development of plants. Plants can elongate their roots and shoots by obtaining sufficient ammonia from endophytes [42]. In addition, ammonia can indirectly stimulate plant growth by inhibiting plant pathogens [43]. In this study, up to 42 isolated endophytes demonstrated varying degrees of ammonia production when Nessler's reagent was added to the broth media (Fig. 1). The endophytic yeast *Cryptococcus laurentii* CY.OS 08 exhibited the highest ammonia production activity, measuring 6.39 ± 0.35 mg/g of dry cell weight (Fig. 2). Since ammonia is a good source of nitrogen for plants, this isolate can provide a nitrogen source for plant growth and increase yields [44].

### Co-inoculation of Two Selected Endophytic Fungi Enhanced Chili Growth

Plant growth-promoting endophytic fungi, including yeasts and molds, have been shown to stimulate plant growth by producing secondary metabolites, such as phytohormones (gibberellin and IAA) [45, 46], siderophore [15] and mineral-solubilizing metabolites [47]. These findings are in accordance with our observation (Table S1). Research by Suryanto D,Bungsu A,Taniwan S,Nurwahyuni I, Pangastuti A [48] has shown that the combination of *Bacillus* sp. (a phosphate solubilizer and IAA producer) and rhizospheric actinomycetes Sp10R (an IAA producer) improved plant performance and inhibited plant pathogen, *Fusarium oxysporum*. However, only a few studies have investigated how combinations of endophytic fungi affect plant growth promotion. In the present study, the effects of single and mixed inoculation of two selected endophytic fungi on chili plants were investigated under pot-scale conditions.

In this case, we co-inoculated chili plants with endophytic strains of mold and yeast. On the germination and growth of chili plants, the effects of a combination of endophytic fungal isolates were determined. The endophytic fungal isolates *A. pullulans* CY.OS 13 and *A. tamarii* CY.OS 144 were used. Both strains, particularly *A. pullulans* CY.OS 13, were capable of producing phytohormones (IAA and GA3) (Table 2). They also demonstrated an antagonistic effect against plant pathogens [19]. As a result, *A. pullulans* CY.OS 13 has been chosen in the present study as a phytohormone-producing fungus. *A. tamarii* CY.OS 144 was considered a potent nutrient solubilizer and producer due to its wide range of activities such as phosphate solubilization, zinc solubilization, siderophore synthesis, and especially ammonia production (Table 2) when compared to other endophytic fungal isolates (Table S1).

The following six treatments were used in this experiment: (T1) seeds treated with sterile-distilled water as a control, (T2) seeds treated with 1 × 10^5^ cells/ml of *A. pullulans* CY.OS 13, (T3) seeds treated with 1 × 10^8^ cells/ml of *A. pullulans* CY.OS 13, (T4) seeds treated with 1 × 10^5^ spores/ml of *A. tamarii* CY.OS 144, (T5) seeds treated with 1 × 10^8^ spores/ml of *A. tamarii* CY.OS 144 and (T6) seeds treated with the combination of 1 × 10^8^ cells/ml of *A. pullulans* CY.OS 13 and 1 × 10^8^ spores/ml of *A. tamarii* CY.OS 144.

**Fig. 3:**
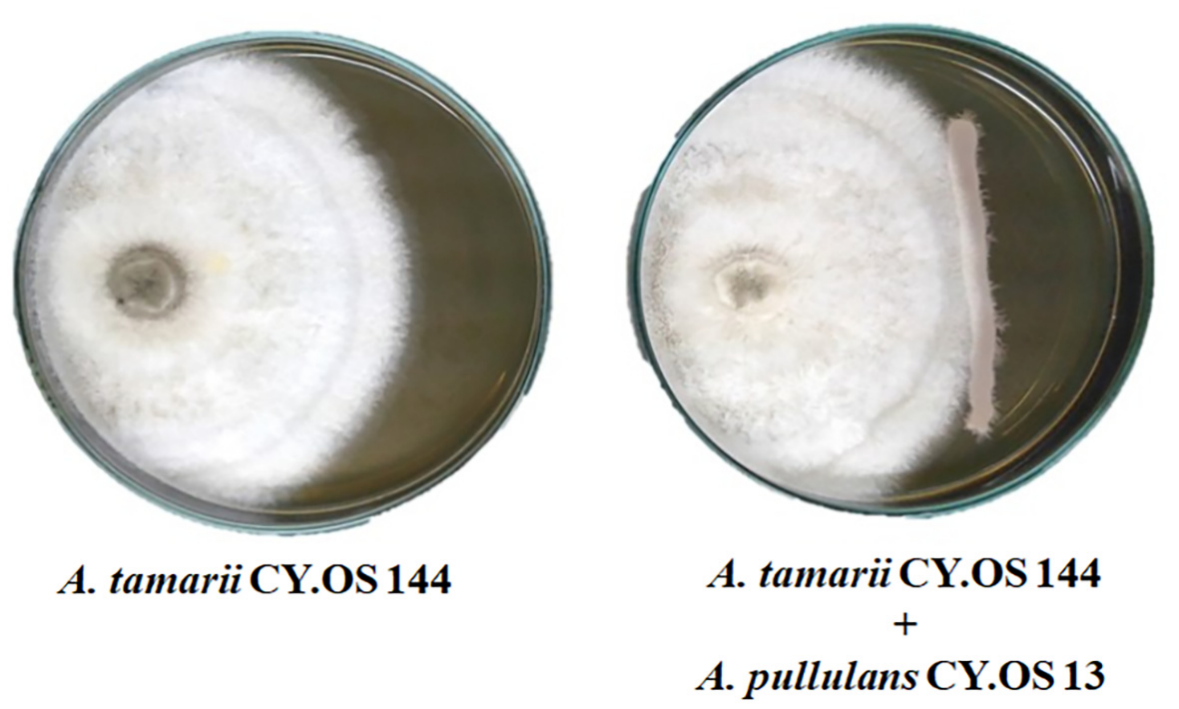
Antagonistic test of selected endophytic fungi using dual culture method. Left: the growth of *A. tamarii* CY.OS 144 as the control, Right: the growth of *A. tamarii* CY.OS 144 against *A. pullulans* CY.OS 13.

**Two selected endophytic fungi showed no antagonistic interaction.** The antagonist activity between two identified endophytic fungi, *A. pullulans* CY.OS 13 and *A. tamarii* CY.OS 144, was evaluated using the dual culture method to determine the effects of their combination on each other’s growth inhibition. The mycelial disc of an actively growing *A. tamarii* CY.OS 144 was placed on the edge of the plate. The *A. pullulans* CY.OS 13 was streaked orthogonally in a line away from the disc. In the following step, the inhibition of mycelial growth was observed. Despite the unclear inhibitory effect of *A. tamarii* CY.OS 144 on a PDA-type test plate (data not shown), our results on the YPD plate demonstrated that the selected endophytic fungi could develop normally and did not interfere with each other's growth (Fig. 3). Consequently, the combination of these endophytic fungi could possibly be investigated in the future.

**Fig. 4:**
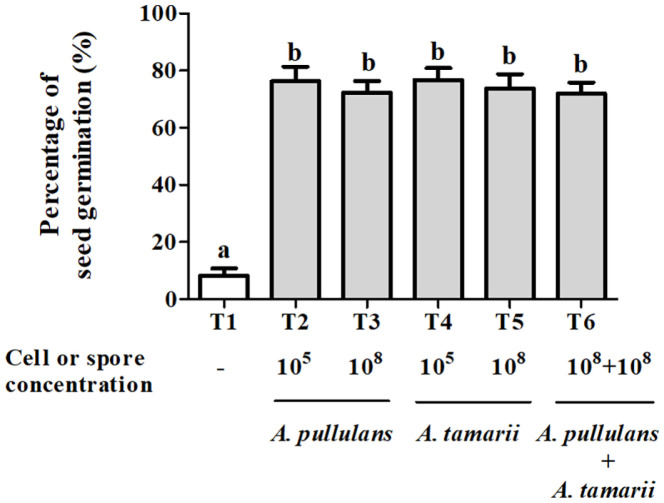
The effect of selected endophytic fungi on chili seed germination by TP method. Data are represented as Mean ± SEM. Different letters indicate significant differences between treatments (*p* < 0.05) based on Tukey’s test. The experiment was planned as a completely randomized design (CRD) with 4 replicates for each treatment (N = 400/treatment).

**Single and co-inoculations of two selected endophytic fungi could improve chili seed germination.** Previous studies have shown that endophytes producing IAA and/or GA3 significantly enhanced seed germination and growth, and also increased the shoot and root length of plants [29, 49]. In this study, the TP method was used to evaluate the effect of selected endophytic fungi on chili seed germination. In line with the findings of previous studies, seeds treated with a single endophyte or a combination of the selected endophytes showed a statistically significant increase in germination of over 60% after ten days of incubation, compared to the control (*p* < 0.001)(Fig. 4). However, no significant difference was observed between the seedlings infected with various concentrations of endophytes. The results indicated that the application of a single endophytic fungus or a combination of selected endophytic fungi to chili seeds could stimulate germination.

**Co-inoculation of selected endophytic fungi could stimulate chili growth under pot-scale conditions.** To investigate the effect of selected endophytic fungi on the growth promotion of chili plants, various plant growth parameters including shoot and root length, biomass, and chlorophyll content were determined. Visual observations showed that the height of chili plants varied among treatments for 30 days after seedling emergence.

**Fig. 5:**
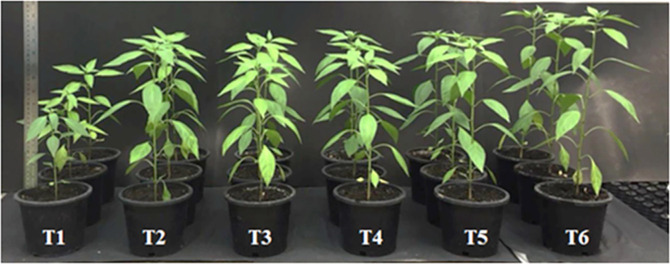
The growth of chili plants in each treatment after 30 days of planting under pot scale condition.

**Fig. 6:**
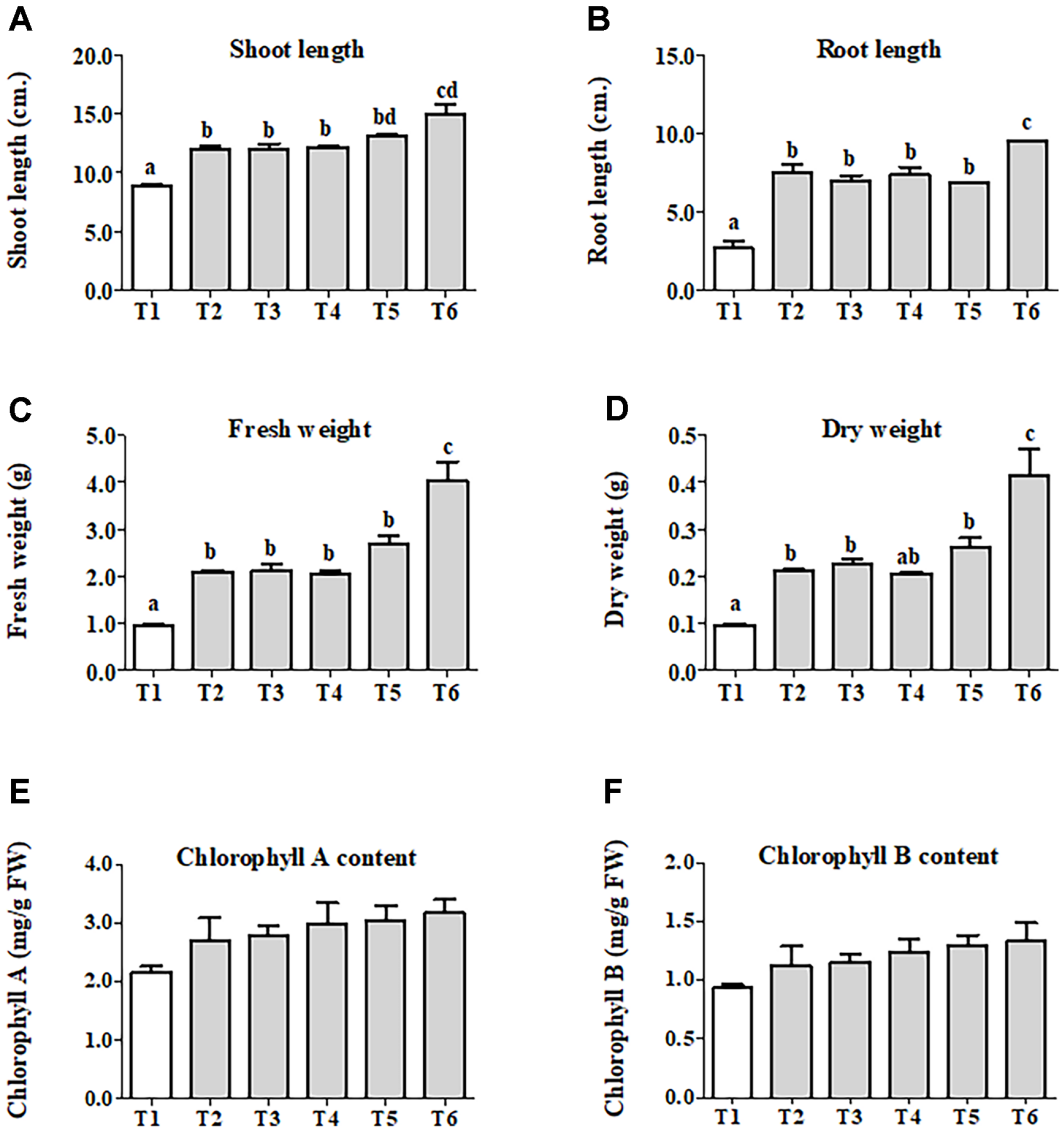
The effects of selected endophytic fungi on promoting chili growth under pot scale condition. Growth characteristics including shoot length (**A**) root length (**B**) fresh weight (**C**) dry weight (**D**) chlorophyll A content (**E**) and chlorophyll B content (**F**) of all treatments are shown. Data are represented as Mean ± SEM. Different letters indicate significant differences between treatments (*p* < 0.05) based on Tukey’s test. The experiment was planned as a completely randomized design (CRD) with 3 replicates for each treatment (N = 30/treatment).

When compared with the uninoculated treatment T1, it was found that the single and especially the co-inoculation treatments significantly increased the height of the chili plants (T2-T6) (Fig. 5). In this experiment, the application of either *A. pullulans* CY.OS 13 or A. tamari CY.OS 144 to the seeds resulted in significantly longer shoots (between 1.3 and 1.7-fold) and root parts (between 2.6 and 3.6-fold) compared to those treated with water controls (T1) (Fig. 6A and 6B). Similarly, the presence of these endophytic fungi led to a significant over 2-fold increase in the fresh and dry biomass of chili plants compared to the control T1 (Fig. 6C and 6D). Chlorophyll concentration, an important parameter for measuring plant growth [32], was determined in fresh chili leaf extract treated with endophytes and in untreated samples. Fig. 6E and 6F shows that among the treatments, endophytic fungi slightly improved both chlorophyll A and B contents when compared with the control T1. It was observed, however, that the cell or spore concentrations of the single inoculation of the endophytic fungus had no significant effect on all parameters (shoot length, root length, fresh and dry weights, chlorophyll A and chlorophyll B contents) (Fig. 6; T2 vs T3 and T4 vs T5). This indicates that the stimulation of chili growth was independent of cell or spore concentrations. Consequently, for the dual inoculation, a high cell or spore concentration was chosen. Compared to single inoculation, dual inoculation significantly improved most of these parameters, except for chlorophyll contents (Fig. 6; T3 vs T6 and T5 vs T6). The plants showed maximum increase in shoot length (1.7-fold), root length (3.6-fold), cell biomass (4-fold), and chlorophyll content (1.4-fold) when dually inoculated with the endophytes *A. pullulans* CY.OS 13 and *A. tamarii* CY.OS 144, compared to plants treated with water controls.

Overall, the findings suggest that the combination of phytohormone-producing fungus *A. pullulans* CY.OS 13 and nutrient-solubilizing fungus *A. tamarii* CY.OS 144 could be beneficial for enhancing plant growth. Consistent with earlier studies, co-inoculation of different microorganisms could enhance the availability and uptake of nutrients, as well as the capacity to produce phytohormones for plants. This can lead to beneficial effects on plant growth, development, and yield [50-53].

**Fig. 7:**
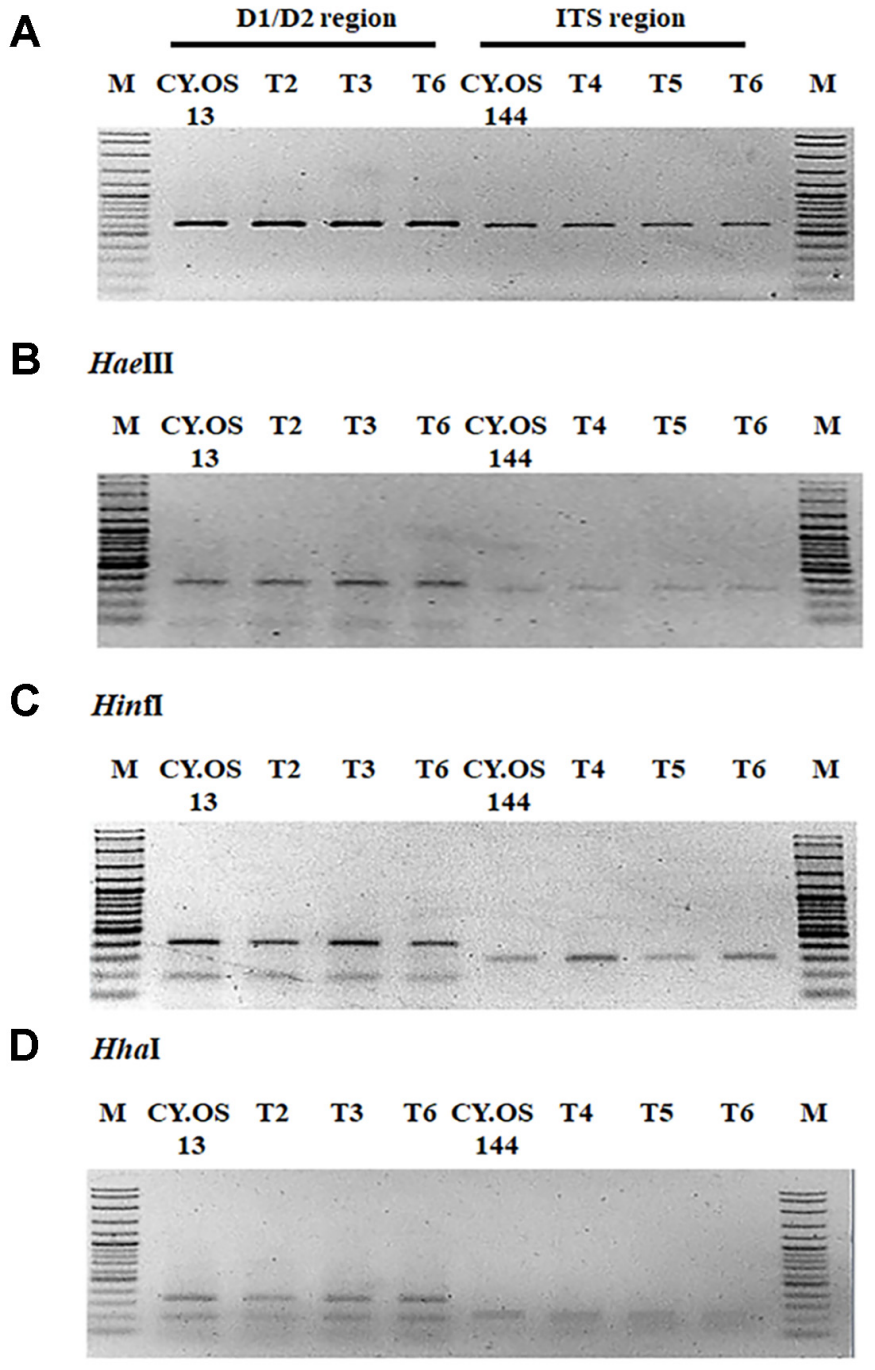
PCR-RFLP profile of either D1/D2 or ITS digested by *Hae*III, *Hin*fI, and *Hha*I. (**A**) PCR products of D1/ D2 and ITS regions. (**B**), (**C**) and (**D**) PCR products digested with designated restriction enzymes. Lanes 1-4 correspond to PCR-RFLP profile of D1/D2 region; lanes 5-8 correspond to PCR-RFLP profile of ITS region. M corresponds to 100 bp DNA ladder.

**Successful colonization of the selected endophytic fungi into chili plants.** The colonization of the selected endophytic fungi, *A. pullulans* CY.OS 13 and *A. tamarii* CY.OS 144, was determined through molecular characterization. PCR-RFLP is a commonly used technique for identifying fungal species [54, 55]. It has various advantages over traditional sequencing, such as simplicity, speed, and accuracy. PCR-RFLP allows for the amplification of a conserved region of DNA sequence using PCR and the detection of genetic variations in species by digesting the amplified fragment with restriction enzymes [56, 57]. The pure cultures of fungal endophyte-inoculated chili plant roots were isolated and grouped. Thereafter, fungal isolate with the same morphology as the selected endophytic fungi was used in the process. For the details of PCR-RFLP analysis in this experiment, the genomic DNA of the endophytic fungus was first isolated and used as a DNA template to amplify the specific D1/D2 region of the large subunit of rDNA or the ITS region of rDNA using pairs of universal primers. Then, the indicated restriction enzymes, *Hae*III, *Hin*fI, and *Hha*I, were used to digest the PCR-amplified DNA fragment to produce a specific DNA fragment pattern. By utilizing the PCR-RFLP assay, *A. pullulans* CY.OS and *A. tamarii* CY.OS 144 served as controls for PCR-RFLP profiles. Our results showed that the PCR-RFLP patterns of the isolated endophytic fungi in the T2, T3, and T6 treatments were identical to those of *A. pullulans* CY.OS 13. Likewise, the treatments T4, T5, and T6 were identical to those used for *A. tamarii* CY.OS 144 (Fig. 7). Hence, the results indicated the successful colonization and entry of fungal endophytes into the plants.

## Conclusion

This study found that endophytic fungi isolated from mango trees showed varying degrees of capacity to promote plant growth. This was demonstrated by their ability to solubilize phosphate, potassium, and zinc, as well as their capacity to produce ammonia and siderophores. We selected *A. pullulans* CY.OS 13 and *A. tamarii* CY.OS 144 as the optimal isolates to investigate the effects of individual and combined inoculation on chili growth, based on their endophytic abilities. Seed inoculation, especially dual inoculation, with selected endophytic fungi has been shown to enhance seed germination, biomass, shoot and root length, and chlorophyll content without any negative effects on host plants. In this study, we demonstrated that a combination of fungal endophytes can be applied to improve crop yield, as they are generally accepted as human and environmentally friendly options. Future research is required to evaluate the effects of the selected endophytic fungi on the growth-promoting activity of other economically important agricultural crops, as well as the ability of endophytic fungi to mitigate the adverse effects of abiotic stress.

## Supplemental Materials

Supplementary data for this paper are available on-line only at http://jmb.or.kr.



## Figures and Tables

**Table 1:** Endophytic fungi isolated from *Mangifera indica* L used in this study.

Endophytic fungi	Isolate code (CY.OS)	Activities
*Aureobasidium pullulans*	01, 02, 03, 10, 11, 13	Antifungal and phytohormone production
*Candida tropicalis*	04	Phytohormone production
*Hanseniaspora opuntiae*	05, 06, 12	Antifungal and phytohormone production
*Candida* sp.	07, 09	Antifungal and phytohormone production
*Cryptococcus laurentii*	08	Phytohormone production
*Colletotrichum gloeosporioides*	16, 17, 21, 64, 111, 113, 114, 122, 140, 161, 162, 164, 209	Antifungal and/or phytohormone production
*Pseudofusicoccum adansoniae*	75, 81, 112, 132, 133, 134, 150, 160, 197, 200, 217	Antifungal and/or phytohormone production
*Aspergillus tamarii*	102, 106, 135, 144, 149, 153, 203, 211, 213, 214, 223	Antifungal and/or phytohormone production
*Penicillium citrinum*	145, 185	Phytohormone production

**Table 2:** Plant growth promoting activities of two selected endophytic fungi.

Endophytic fungi isolate	Plant growth promoting activities					
	IAA production (mg/ g DCW)	GA3 production (μg/ g DCW)	Siderophore production (PE)	Phosphate solubilization (SE)	Zinc solubilization (SE)	Ammonia production (mg/ g DCW)
*A. pullulans* CY.OS 13	11.01 ± 0.47	1.37 ± 0.25	-	-	-	2.86 ± 0.68
*A. tamarii* CY.OS 144	2.10 ± 0.79	-	1.29 ± 0.11	1.08 ± 0.04	0.80 ± 0.10	1.77 ± 0.41

Data are represented in Mean ± SEM and all experiments were performed in triplicate.

DCW: Dry cell weight.

- : No activity
