# Supplementary material for: Roles of Endophytic Fungi Isolated from Mangifera indica L. in Promoting Plant Growth
Source: J Microbiol Biotechnol. 2024 Jul 19;34(9):1857–66. doi: 10.4014/jmb.2401.01034 (PMC11473608; doi:10.4014/jmb.2401.01034)
Supplement: Supplementary file 1 [file jmb-34-9-1857-supple.pdf]

J. Microbiol. Biotechnol. 2024. 34(9): 1857-1866

https://doi.org/10.4014/jmb.2401.01034

Supplementary Tables

Table S1. Plant growth promoting activities of endophytic fungi.

Endophytic fungi	Isolates	Phosphate solubilization efficiency (SE)	Potassium solubilization efficiency (SE)	Zinc solubilization efficiency (SE)	Siderophore production efficiency (PE)	Ammonia production (mg/g DCW*)
Aureobasidium pullulans	CY. OS 01	1.09±0.04	-	-	-	4.49±0.66
Aureobasidium pullulans	CY. OS 02	1.30±0.13	-	-	-	3.66±0.68
Aureobasidium pullulans	CY. OS 03	1.19±0.06	-	-	-	3.61±0.79
Candida tropicalis	CY. OS 04	1.11±0.02	-	-	-	4.14±0.38
Hanseniaspora opuntiae	CY. OS 05	-	-	-	-	-
Hanseniaspora opuntiae	CY. OS 06	-	-	-	-	-
Candida sp.	CY. OS 07	1.61±0.19	1.38±0.04	-	0.94±0.04	0.40±0.07
Cryptococcus laurentii	CY. OS 08	1.18±0.09	-	-	-	6.39±0.35
Candida sp.	CY. OS 09	1.31±0.10	1.16±0.05	-	1.13±0.03	2.46±0.36
Aureobasidium pullulans	CY. OS 10	1.37±0.16	-	-	-	4.47±0.72

Aureobasidium pullulans	CY. OS 11	-	-	-	-	2.65±0.53
Hanseniaspora opuntiae	CY. OS 12	-	-	-	-	-
Aureobasidium pullulans	CY. OS 13	-	-	-	-	2.86±0.68
Colletotrichum gloeosporioides	CY. OS 16	-	-	-	1.09±0.01	1.37±0.15
Colletotrichum gloeosporioides	CY. OS 17	-	-	-	1.08±0.02	1.51±0.04

Table S1. Plant growth promoting activities of endophytic fungi (Cont.).

Endophytic fungi	Isolates	Phosphate solubilization efficiency (SE)	Potassium solubilization efficiency (SE)	Zinc solubilization efficiency (SE)	Siderophore production efficiency (PE)	Ammonia production (mg/g DCW*)
Colletotrichum gloeosporioides	CY. OS 21	-	-	-	1.08±0.01	1.32±0.16
Colletotrichum gloeosporioides	CY. OS 64	-	-	-	1.08±0.01	1.40±0.12
Pseudofusicoccum adansoniae	CY. OS 75	-	-	-	1.00±0.00	0.39±0.05
Pseudofusicoccum adansoniae	CY. OS 81	-	-	-	1.00±0.00	0.51±0.05
Aspergillus tamarii	CY. OS 102	1.02±0.06	-	1.10±0.08	2.36±0.15	1.90±0.08
Aspergillus tamarii	CY. OS 106	-	-	1.04±0.05	2.43±0.00	1.80±0.09
Colletotrichum gloeosporioides	CY. OS 111	-	-	-	1.42±0.32	0.18±0.01

Pseudofusicoccum adansoniae	CY. OS 112	-	-	-	1.03±0.04	0.17±0.01
Colletotrichum gloeosporioides	CY. OS 113	-	-	1.03±0.05	2.36±0.15	1.72±0.10
Colletotrichum gloeosporioides	CY. OS 114	-	-	-	1.00±0.00	0.35±0.12
Colletotrichum gloeosporioides	CY. OS 122	-	-	-	0.89±0.00	1.14±0.09
Pseudofusicoccum adansoniae	CY. OS 132	-	-	-	1.00±0.00	-
Pseudofusicoccum adansoniae	CY. OS 133	-	-	-	1.00±0.00	-
Pseudofusicoccum adansoniae	CY. OS 134	-	-	-	1.00±0.00	-
Aspergillus tamarii	CY. OS 135	-	-	0.55±0.05	2.50±0.35	2.74±0.28

Table S1. Plant growth promoting activities of endophytic fungi (Cont.).

Endophytic fungi	Isolates	Phosphate solubilization efficiency (SE)	Potassium solubilization efficiency (SE)	Zinc solubilization efficiency (SE)	Siderophore production efficiency (PE)	Ammonia production (mg/g DCW*)
Colletotrichum gloeosporioides	CY. OS 140	-	-	-	1.00±0.00	0.41±0.15
Aspergillus tamarii	CY. OS 144	1.08±0.04	-	0.80±0.14	1.29±0.11	1.77±0.41
Penicillium citrinum	CY. OS 145	-	-	2.17±0.12	1.96±0.29	1.69±0.27
Aspergillus tamarii	CY. OS 149	-	-	0.57±0.11	1.28±0.08	1.41±0.23
Pseudofusicoccum adansoniae	CY. OS 150	-	-	-	0.89±0.00	0.53±0.13
Aspergillus tamarii	CY. OS 153	-	-	1.00±0.00	1.29±0.01	1.59±0.37
Pseudofusicoccum adansoniae	CY. OS 160	-	-	-	1.00±0.00	0.03±0.01
Colletotrichum gloeosporioides	CY. OS 161	-	-	-	0.96±0.03	1.31±0.06
Colletotrichum gloeosporioides	CY. OS 162	-	-	1.50±0.00	1.00±0.00	0.81±0.26
Colletotrichum gloeosporioides	CY. OS 164	-	-	1.00±0.00	-	1.61±0.08
Penicillium citrinum	CY. OS 185	-	-	1.00±0.00	4.60±0.30	1.17±0.06
Pseudofusicoccum adansoniae	CY. OS 197	-	-	-	1.11±0.01	1.17±0.21
Pseudofusicoccum adansoniae	CY. OS 200	-	-	-	1.08±0.01	1.08±0.27

Aspergillus tamarii	CY. OS 203	-	-	1.00±0.00	1.27±0.07	-
Colletotrichum gloeosporioides	CY. OS 209	-	-	-	1.17±0.04	1.17±0.23

Table S1. Plant growth promoting activities of endophytic fungi (Cont.).

Endophytic fungi	Isolates	Phosphate solubilization efficiency (SE)	Potassium solubilization efficiency (SE)	Zinc solubilization efficiency (SE)	Siderophore production efficiency (PE)	Ammonia production (mg/g DCW*)
Aspergillus tamarii	CY. OS 211	1.00±0.00	-	0.53±0.11	1.55±0.18	0.06±0.03
Aspergillus tamarii	CY. OS 213	-	-	0.60±0.09	1.00±0.00	-
Aspergillus tamarii	CY. OS 214	1.03±0.01	-	1.07±0.09	2.29±0.06	0.01±0.00
Pseudofusicoccum adansoniae	CY. OS 217	-	-	-	1.00±0.00	0.88±0.14
Aspergillus tamarii	CY. OS 223	1.00±0.00	-	1.03±0.02	1.34±0.04	0.02±0.01

The data showed only the strains exhibiting phosphate solubilization, potassium solubilization, zinc solubilization, siderophore production and ammonia production activities.

Data represents Mean ± SEM. The experiments were performed in triplicate.

*DCW= Dry cell weigh
